# Cases of Eccentric Epilepsy, Etc.

**Published:** 1876-05

**Authors:** W. R. McMahan

**Affiliations:** Huntingburg, Ind.


### CASES OF ECCENTRIC EPILEPSY FROM IRRITATION OF THE GLANS PENIS.

By W. R. McMAHAN, M.D., Huntingburg, Ind.

The causative agent in each given case primarily is either centric or eccentric. In the former we suppose that there is, and has been, some subtle molecular change in the nerve cells in the region of the base of the brain, and this change is of such a nature, degree and intensity as to assume the full rounded form of a cause, fully adequate to the production of epilepsy. But it is to the eccentric causes of epilepsy that I wish to direct attention; and here we find that we are not thrown, in all cases, on the unguided drift of supposition, as in the former (centric causes); but that we can often localize and demonstrate the causative agent, and, as such, disrobe it of its power, or remove it from the temple it is despoiling.

But we will proceed to the enumeration of the stereotyped eccentric causes, as follows: "Mechanical injuries to some sentient nerve, or its inclusion in the contraction of a cicatrix; irritation of the intestinal canal from worms or indigestible matters; and a morbid condition of the genital organs." These, in their broadest sense, constitute the principal eccentric causes. "Cases arising from the first enumerated cause may occur at any age." Cases arising from the second have been, and I think rightly too, confined to the age of childhood; but the cases arising from a morbid or irritated condition of the genital organs have been, as I conceive, erroneously confined to the age of puberty, and excluded as a causative agent in the epilepsy of children, where it yields a large percentum of cases.

My attention was first called to this subject by the report of Prof. Sayre, to the American Medical Association, of a number of cases of reflex paralysis caused by the irritation arising from phymosis and adherent prepuce to the corona of the organ. And then, reasoning from analogy, I came to the following conclusions: That if this abnormal condition of the genital organs could, and had, produced, in a given number of cases, anæmia of the spinal cord, and, as an effect of that, paralysis of the inferior extremities, the same cause might, and most probably would, in a certain number of cases, produce epilepsy, or any other nervous manifestation that is susceptible of having an eccentric origin.

Again, Sir Thomas Watson, in speaking of the causes of epilepsy, enumerates: debauchery of all kinds; the habitual indulgence in intoxicating liquors; and, above all, the most powerful predisposing cause, not congenital, is masturbation. Sir Charles Locock attributes the great increase of the disease, during late years, to the last named cause.

Now, if these opinions of these great men are correct, that masturbation will produce irritation of the sentient nervous system sufficient to produce epilepsy in the adult, then we are forced to the conclusion that irritation of the genital organs, in a greatly diminished degree, would produce epilepsy in childhood, a time when the over-sensitive nervous system stands trembling on the brink of convulsions, only awaiting a slight jar or nervous discord to precipitate an attack. Now, the irritation arising from phymosis, or the accumulation of smegma behind the adherent bands of the prepuce in the male, or from an abnormal condition of the genital organs in the female, producing a degree of irritation equal in intensity to that of phymosis or adherent prepuce in the male, is, in my opinion, fully adequate to produce epilepsy in childhood.

The foregoing conclusion I consider verified in the following two cases:

Case I. C. D., a well-developed, round-faced, good-looking boy. His parents state that he was in good health until January, 1874, when, without any premonitions whatever, he was seized with a severe convulsion, characterized by perfect loss of consciousness; a contracted, trembling condition of voluntary muscles; grinding of teeth, and foaming at the mouth. This condition lasted from one to two minutes, when relaxation commenced, which merged into stupor of several hours duration, when he awoke rational. His penis was now noticed to be erected, and on examination (by his parents), found in an irritated condition. He had several seizures during the first two days, and then none for several weeks, when the cycle was repeated.

This order of activity and repose continued unabated under the bromides and various other forms of treatment, until April, 1875, at which time, his parents state, his penis was in a state of irritation, and that considerable pus escaped from the preputial orifice, after which his convulsions ceased in the main, leaving only a light "*Petit mal*" variety, separated by long intervals.

On December 22, 1875, I examined his genital organs, and found a long redundant prepuce, and partial phymosis. The prepuce was retracted over the corona with great difficulty. After its retraction, the crease behind the corona was found in a suppurating condition. On the sides of the corona were suppurating spots, marking the site of former adhesions of the prepuce to the corona. The entire crown of the organ was in a red, irritated condition, but contained no imprisoned smegma or pus.

After the examination, I ordered the prepuce retracted once each day, parts cleansed, and then sponged with some mild astringent. At this time his father states that the irritation has subsided, and that there are no symptoms whatever of epilepsy.

The analysis of this case I conceive to be this: At the time of his first seizure, there was either adhesion of the prepuce to the corona of the organ, or the prepuce enveloped the crown so closely that the heretofore accumulated smegma (and perhaps pus) could not make its escape, acted as a foreign body, irritated the sentient nerves, and through these the convulsive centre or centres. That this condition continued until last April, when the adhesions or resistance gave way before the imprisoned matters, and they made their escape; these sentient nerves were no longer teased; the cause was removed, and the boy recovered.

CASE II. Chas. Fisher was attacked with epilepsy on April 7, 1874. Up to that time he was a well-developed, good-looking, bright boy, and enjoyed uninterrupted good health. His attack was of a sudden character, and not preceded by any premonitions; marked by about the same characteristics as the former case, as regards the frequency, duration and intensity of the attacks; and this order continued, in modified form, under large doses of the bromides, and other treatment that seemed to be indicated, until the 27th of December, 1875.

On December 21, 1875, I examined his genital organs, and found firm adhesions just behind the meatus urinarius; and on Dec. 27, 1875, I separated these adhesions and retracted the prepuce, behind which was imprisoned smegma in considerable quantity, and the parts were in a state of irritation.

From the date of his attack until this time, he had gradually grown dull and listless; was clumsy, irascible, and his eye, at times, generally preceding his attack, would assume the appearance of a fish's. His parents state that before, during and after these groups of convulsions, he would complain of his penis, and that it was often in a state of erection. After the breaking up of adhesions over a month ago, all medication was suspended, and at present he is well; has not had a single symptom of a convulsion since; has regained his former activity, and his eye its wonted expressiveness. The change in his condition is so great that I have no fears of a return of his trouble.

I regret that I did not make an ophthalmoscopic examination of his eyes prior to operating, and at a time when his eyes wore a "fish-like" appearance. This appearance of these organs must have depended on a departure of the circulation from a normal standard, dependent on disturbance of the sympathetic system.

